# Epidemiological and clinical factors associated with post-exertional malaise severity in patients with myalgic encephalomyelitis/chronic fatigue syndrome

**DOI:** 10.1186/s12967-020-02419-4

**Published:** 2020-06-22

**Authors:** Alaa Ghali, Paul Richa, Carole Lacout, Aline Gury, Anne-Berengere Beucher, Chadi Homedan, Christian Lavigne, Geoffrey Urbanski

**Affiliations:** 1 Department of Internal Medicine, University Hospital, 49000 Angers, France; 2 Department of Biochemistry and Genetics, University Hospital, 49000 Angers, France

**Keywords:** Myalgic encephalomyelitis/chronic fatigue syndrome, Post-exertional malaise, Age, Recurrent infections, Infectious precipitants

## Abstract

**Background:**

Post-exertional malaise (PEM), the cardinal feature of myalgic encephalomyelitis/chronic fatigue syndrome (ME/CFS), occurs generally after exposure to a stressor. It is characterized by the worsening of ME/CFS symptoms and results in aggravating the course of the disease and the quality of life of patients. Due to its unpredictable onset, severity, and recovery time, identifying patients with higher risk for severe PEM would allow preventing or reducing its occurrence. We thus aimed at defining possible factors that could be associated with PEM severity.

**Methods:**

Adult patients fulfilling ME international consensus criteria who attended the internal medicine department of University hospital Angers-France between October 2011 and December 2019 were included retrospectively. All patients were systematically hospitalized for an etiological workup and overall assessment. We reviewed their medical records for data related to the assessment: epidemiological data, fatigue features, clinical manifestations, and ME/CFS precipitants. PEM severity was appreciated by the Center for Disease Control self-reported questionnaire. The study population was classified into quartiles according to PEM severity scores. Analyses were performed with ordinal logistic regression to compare quartile groups.

**Results:**

197 patients were included. PEM severity was found to be positively associated with age at disease onset ≥ 32 years (OR 1.8 [95% CI 1.1–3.0] (p = 0.03)), recurrent infections during the course of the disease (OR 2.1 [95% CI 1.2–3.7] (p = 0.009)), and when ME/CFS was elicited by a gastrointestinal infectious precipitant (OR 5.7 [1.7–19.3] (p = 0.006)).

**Conclusion:**

We identified some epidemiological and clinical features, which were positively associated with PEM severity in subsets of ME/CFS patients. This could help improving disease management and patients’ quality of life.

## Background

Myalgic encephalomyelitis also known as chronic fatigue syndrome (ME/CFS) is a long-term and debilitating multisystem condition of unknown etiology affecting several millions of individuals worldwide [1]. It is characterized by persistent or relapsing unexplained fatigue of more than 6 months’ duration that is not alleviated by rest and associated with a variety of symptoms, especially unrefreshing sleep, cognitive difficulties, orthostatic intolerance, and pain. Nevertheless, the post-exertional malaise (PEM) is the cardinal feature of ME/CFS, and recent diagnostic criteria require its presence [2–4]. It helps to distinguish ME/CFS from idiopathic chronic fatigue [3], and other diseases with chronic fatigue such as multiple sclerosis [5], depression [6], and systemic lupus erythematosus [7]. PEM is not just generalized fatigue, but an abnormal worsening of a patient’s baseline symptoms after exposure to physical or cognitive stressors that were normally tolerated before disease onset [3, 4]. The type, intensity, and frequency of PEM symptoms are often unexpected and out of proportion to the type, intensity, frequency, and duration of the PEM stressor [4].

Several PEM stressors were reported, especially physical or cognitive exertion [8], emotional distress [9], infections [4, 10], exposure to chemicals [11], physical trauma, and sleep debt [4]. PEM onset is unpredictable since it may occur immediately after stressor or delayed by several hours or days. Similarly, PEM duration varies largely not only between patients but also within the same patient and over the course of the disease. This variation is probably at least partially dependent on the baseline state of individuals such as for instance the level of exertion involved several days before PEM onset or the presence of infection [9]. The pathophysiology of PEM is not yet well understood. However some studies showed that mitochondrial dysfunction may have a role in PEM nature [12, 13]. We recently reported that elevated blood lactate levels at rest in a subgroup of ME/CFS patients were associated with more severe PEM [14].

In the absence of ME/CFS treatment and given the fact that PEM was found to be significantly associated with disability [15] and predict a poorer outcome for patients [16], the prevention of its occurrence, or at least reducing its severity, is very important to prevent disease exacerbation. Our study aimed at identifying possible epidemiological and/or clinical factors that could be associated with PEM severity.

## Methods

The study was approved by the ethics committee of the University hospital of Angers (2018/46) and was conducted in compliance with the Helsinki agreement.

We reviewed all medical records of patients attending the outpatient clinic of the internal medicine department of Angers University Hospital and diagnosed as having ME/CFS between October 1, 2011 and December 31, 2019. The diagnosis of ME/CFS was established by the same physician. We enrolled all patients aged ≥ 18 years who met the ME International Consensus Criteria (ME ICC) 2011 [3]. In accordance with these criteria, patients with an identifiable medical condition that could account for chronic fatigue, and those with primary psychiatric disorders or substance dependence were excluded. According to ME ICC, comorbidities such as fibromyalgia, irritable bowel syndrome (IBS), Hashimoto’s thyroiditis, and reactive depression did not constitute an exclusionary condition. Epidemiological data, fatigue features, ME/CFS precipitants, and comorbidities were collected for each patient. Patterns of symptom clusters according to the ME ICC criteria were carefully extracted [3].

Fatigue level was assessed by means of validated self-reported questionnaires; the fatigue scale (FS) [17] and the fatigue severity scale (FSS) [18]. The impact of fatigue on patient activities was assessed by the modified fatigue impact scale (MFIS) [19].

PEM severity over the past month was assessed by means of the PEM item from the standardized self-reported questionnaire of Center for Disease Control and Prevention Symptom Inventory (CDC SI) [20]. Perceived frequency of PEM was rated on a 4-point scale (1 = a little of the time, 2 = some of the time, 3 = most of time, 4 = all of the time), and its intensity was measured on a 3-point scale (1 = mild, 2 = moderate, 3 = severe). The intensity score was converted into equidistant score (0 = symptom not reported, 1 = mild, 2.5 = moderate, 4 = severe). The frequency and intensity scores were then multiplied to create the PEM severity score ranging from 0 to 16.

In the absence of a validated threshold defining PEM severity, patients were grouped into quartiles (Q) according to PEM severity scores of the studied population: Q1 (mild PEM with scores ≤ 5); Q2 (moderate PEM with scores 7.5, 8, and 10); Q3 (severe PEM with score = 12), and Q4 (very severe PEM score = 16).

Qualitative data were expressed as an absolute number and percentage. Quantitative data were expressed as median and quartiles. The alpha risk was set at 5%. The odds ratio (OR) was presented with its 95% confidence interval (CI). Analyses were performed with ordinal logistic regression to compare the 4 quartile groups. All covariates of a domain were included in the first model for each analysis. We rejected models that did not satisfy validation conditions for ordinal regression: (i) model has to differ from the intercept based on the Chi2-square test on -2log-likelihood ratio (p value < 0.05) and (ii) model has to validate the proportional odds assumption with no difference on the parallel lines test (p-value > 0.05). The final model was created by removing variables one by one with a descending stepwise method and was the first one to validate both the test of parallel lines and the test on -2log-likelihood ratio. The analyses were performed using SPSS software v23.0 (IBM Corp).

## Results

Among the 203 patients who were fulfilling inclusion criteria, we excluded 6 patients in whom data concerning PEM assessment were missing in their medical records, so 197 patients were included in the study. The whole population had a median age at disease onset of 32 [25–40] years, a median body mass index of 22.8 [20.0–25.9] kg/m^2^, and a male to female ratio of 1:2.9. The median delay in diagnosis was 47 [22–102] months. Table 1 provides a descriptive representation of the study population characteristics according to quartiles of PEM severity. Fatigue scales showed high levels of fatigue and fatigue-related impairment with no differences between PEM severity groups.

**Table 1:** Characteristics of patients according to post-exertional malaise severity quartiles

	Quartile 1 mild PEM^a^	Quartile 2 moderate PEM	Quartile 3 severe PEM	Quartile 4 very severe PEM
Epidemiological data				
Patients, n (%)	23 (11.7)	74 (37.6)	73 (37.1)	27 (13.7)
Females, n (%)	17 (73.9)	58 (78.4)	50 (68.5)	21 (77.8)
Basal metabolic index, kg/m^2^	23.7 [20.2–26.3]	23.3 [20.3–25.9]	22.5 [19.6–25.9]	21.9 [20.1–24.6]
Age at disease onset, years	28 [21–38]	32 [25–41]	32 [27–39]	38 [31–41]
Delay in diagnosis, months	60 [25–108]	62 [29.6–120]	44 [21–95]	26 [17–54]
Fatigue features				
Fatigue severity scale	5.4 [5–6] (n = 18)	5.5 [5.03–6.2] (n = 58)	5.9 [5.15–6.5] (n = 59)	5.9 [5.2–6.7] (n = 17)
Fatigue scale	24 [18–27.5] (n = 15)	21 [18–25.5] (n = 59)	25 [22–28] (n = 61)	26 [22–28] (n = 21)
MFIS^b^ physical	29.5 [26.5–32] (n = 16)	28 [24.3–31.8] (n = 58)	31 [27–33] (n = 59)	30 [26.5–33] (n = 22)
MFIS cognitive	24 [19–34] (n = 16)	26 [19.2–30] (n = 58)	31 [24.5–33.5] (n = 59)	26 [22.3–30.6] (n = 22)
MFIS psychosocial	6 [5–7] (n = 16)	5 [4–6] (n = 58)	6 [5–8] (n = 59)	6 [5–7] (n = 22)
Symptom clusters^c^, n (%)				
Neurological impairments	23 (100)	74 (100)	73 (100)	27 (100)
Neurocognitive impairments	23 (100)	74 (100)	73 (100)	27(100)
Difficulty processing information	23 (100)	68 (91.9)	70 (95.9)	27 (100)
Short-term memory loss	16 (69.6)	62 (83.8)	66 (90.4)	24 (88.9)
Pain	22 (95.7)	72 (97.3)	71 (97.3)	26 (96.3)
Headaches	16 (69.6)	53 (71.6)	56 (76.7)	21 (77.8)
Significant pain	19 (82.6)	64 (86.5)	68 (93.2)	26 (96.3)
Sleep disturbances	22 (95.7)	74 (100)	72 (98.6)	27 (100)
Disturbed sleep patterns	21 (91.3)	65 (87.8)	64 (87.7)	22 (81.5)
Unrefreshed sleep	21 (91.3)	74 (100)	69 (94.5)	25 (92.6)
Neurosensory, perceptual and motor disturbances	22 (95.7)	73 (100)	73 (100)	27 (100)
Neurosensory and perceptual troubles	20 (87)	62 (83.8)	69 (94.5)	23 (85.2)
Motor disturbances	20 (87)	69 (93.2)	69 (94.5)	27 (100)
Immune, gastrointestinal, and genitourinary impairments	21 (91.3)	66 (89.2)	71 (97.3)	24 (88.9)
Flu-like symptoms	16 (69.6)	50 (67.6)	59 (80.8)	19 (70.4)
Susceptibility to viral infections	3 (13)	26 (35.1)	31 (42.5)	12 (44.4)
Gastrointestinal impairments	17 (73.9)	63 (85.1)	60 (82.2)	22 (81.5)
Genitourinary impairments	2 (8.7)	28 (37.8)	29 (39.7)	7 (25.9)
Sensitivities to food, medications, odors, or chemicals	2 (8.7)	6 (8.1)	4 (5.5)	35 (11.1)
Energy production/transportation impairments	22 (95.7)	74 (100)	72 (98.6)	27 (100)
Cardiovascular manifestations	19 (82.6)	72 (97.3)	65 (89)	27 (100)
Respiratory manifestations	9 (39.1)	36 (48.6)	38 (52.1)	19 (70.4)
Loss of thermostatic instability	18 (78.3)	65 (87.8)	61 (83.6)	26 (96.3)
Intolerance of extremes of temperatures	13 (56.5)	61 (82.4)	42 (57.5)	21 (77.8)

Categorical data were expressed as absolute number and percentage

Continuous data were expressed as median and quartiles

^a^ Post-exertional malaise

^b^Modified fatigue impact scale

^C^ME International Consensus Criteria [3]

The multivariate ordinal regression analysis of patients’ characteristics showed that only the age at disease onset (≥ 32 years) (OR 1.8 [95% CI 1.1–3.0] (p = 0.03)), and the susceptibility to viral infections (OR 2.1 [95% CI 1.2–3.7] (p = 0.009)) were positively associated with PEM severity (Table 2). The PEM severity groups Q3 (p = 0.03) and Q4 (p = 0.002) were different from the reference Q1 group, but not the Q2 group (p = 0.25).

**Table 2:** Epidemiological and clinical patients’ characteristics associated with post-exertional malaise severity in an adjusted model

	p-value	OR [95% CI]^a^
Post-exertional malaise severity quartiles		
Quartile 1	Reference	
Quartile 2	0.25	
Quartile 3	0.03	
Quartile 4	0.002	
Variables		
Age at onset^b^	0.03	1.8 [1.1–3.0]
Sex (female)	0.30	0.7 [0.4–1.3]
Neurosensory, perceptual and motor disturbances	0.20	6.5 [0.4–108.6]
Sleep disturbances	0.25	4.7 [0.3–65.5]
Susceptibility to viral infections	0.009	2.1 [1.2 –3.7]
Respiratory manifestations	0.06	1.7 [1.0–2.9]
Loss of thermostatic instability	0.24	1.6 [0.7–3.7]

Multivariate analysis was performed with ordinal regression. All variables including age at disease onset, sex, and symptom clusters of ME ICC criteria [3] were included in the initial model. The initial model did not meet the validation conditions: the model significantly differed from the intercept (p = 0.04) but failed to validate the proportional odds assumption (p = 0.015). The final model was created by removing the variables one by one with a descending stepwise method until validation of both the parallel lines test (p = 0.242) and the test on -2log-likelihood ratio from the intercept (p = 0.009)

^a^Odds Ratio with 95% Confidence interval

^b^Age as a categorical variable with a cut-off ≥ the median age of the study population (32 years)

ME/CFS precipitants were identified in 139/197 (70.6%) patients. An infectious event before disease onset was experienced by 97/197 patients (49.2%). Influenza-like illness was the most frequent infectious precipitants in the 4 groups. The distribution of non-infectious and different infectious precipitants of ME/CFS according to quartiles of PEM severity is summarized in Table 3.

**Table 3:** Distribution of ME/CFS precipitants according to post-exertional malaise severity quartiles

	Quartile 1	Quartile 2	Quartile 3	Quartile 4
Patients, n (%)	23 (11.7)	74 (37.6)	73 (37.1)	27 (13.7)
ME/CFS precipitants				
Identified, n (%)	18 (78.3)	46 (62.2)	56 (76.6)	19 (70.4)
Non-infectious, n (%)	4 (17.4)	17 (23)	17 (23)	4 (14.8)
Infectious, n (%)	14 (60.9)	29 (39.2)	39 (53.4)	15 (55.6)
Influenza-like illness	10 (43.5)	13 (17.6)	21 (28.8)	7 (25.9)
Respiratory	0 (0.0)	9 (12.2)	6 (8.2)	3 (11.1)
Gastrointestinal	0 (0.0)	3 (4.1)	3 (4.1)	5 (18.5)
Urinary	0 (0.0)	1 (1.4)	1 (1.4)	0 (0.0)

After adjustment for previously identified variables associated with PEM severity (age at disease onset and susceptibility to viral infections, Table 2), and non-infectious precipitants, gastrointestinal (GI) infectious precipitants were strongly associated with PEM severity (OR 5.7 [1.7–19.3] (p = 0.006)) (Table 4). PEM severity groups Q2, Q3 and Q4 differed from the reference group Q1.

**Table 4:** ME/CFS precipitants associated with post-exertional malaise severity in an adjusted model

Post-exertional malaise severity quartiles	p-value	OR [95% CI]^a^
Quartile 1	Reference	
Quartile 2	< 0.0001	
Quartile 3	0.006	
Quartile 4	< 0.0001	
Variables		
Non-infectious precipitants	0.51	1.3 [0.6–2.6]
Infectious precipitants		
Influenza-like illness	0.30	1.4 [0.7–2.8]
Gastrointestinal	0.006	5.7 [1.7–19.3]
Urinary	0.55	2.2 [0.2–30.2]
Respiratory	0.44	1.5 [0.6–3.8]

Multivariate analysis was performed with ordinal regression. All listed precipitants were included in the initial model and adjusted for previously identified variables (age at disease onset and susceptibility to viral infections). The initial model met the validation conditions: the model differed from the intercept (p = 0.01) and validated the proportional odds assumption (p = 0.23). Age at disease onset (p = 0.03) and susceptibility to viral infections (p = 0.02) remained associated with PEM severity quartiles

^a^Odds Ratio with 95% Confidence interval

## Discussion

PEM constitutes a burden for ME/CFS patients because of its unpredictable onset and severity and due to the fact that, in most cases, it requires a long recovery period. Moreover, studies showed that PEM was associated with disabilities and poorer outcome [15, 16]. The prevention of PEM occurrence or reducing its severity is thus one of the main goals of pacing strategies, which proved successful in ME/CFS management [21]. We thus attempted to determine whether there are factors that could be associated with PEM severity. Results of the current study showed that older age at disease onset, susceptibility to viral infections during the course of the disease, and GI infections prior to disease onset were independently associated with PEM severity.

In the study population, the median age of disease onset of 32 [5–40] years and the higher prevalence of women were comparable to that previously reported [4, 22]. The median time to diagnosis was 47 [22–102] months, which is consistent with the IOM report [4] that showed that time between the onset of symptoms and the diagnosis was longer than 5 years in about a third of patients.

Fatigue assessment showed high median levels of reported fatigue in the whole population with no significant difference between quartiles. This could be explained by the subjective nature of fatigue and the fact that current available fatigue scales would not accurately reflect fatigue severity in chronic fatigue patients [23].

PEM severity was assessed in all patients by the standardized CDC SI self-reported questionnaire [20], which is a reliable and valid instrument for assessing symptoms associated with CFS including PEM, and one of the two tools proposed by the IOM for PEM assessment [4]. CDC SI severity scores reflect both intensity and frequency of PEM and thus offer a means of answering the question of what is more serious: a substantial PEM that appears irregularly or less important PEM that occurs repeatedly. To date, there is still no validated threshold defining PEM severity, so we classified our population into quartiles according to PEM severity.

The first finding of our study is that older age at onset, ≥ 32 years, was positively associated with PEM severity. To the best of our knowledge, the influence of age at ME/CFS onset on PEM severity was not reported before. Nevertheless, there are some evidences that age is linked to the course of the disease. In that respect, increasing age predicted worsening of symptoms with poor prognosis in patients with CFS [24, 25], and an age over 38 years at diagnosis was found to be a risk factor for persistent illness [26]. Jason et al. reported that CFS patients who were older had higher frequencies of symptoms and were more severely disabled [27]. By contrast, Wilson et al. showed that age did not predict outcome in CFS patients [28]. On the other hand, it is generally accepted that young people with ME/CFS have a more favorable prognosis and are more likely to improve and recover compared to adults [29, 30].

Age could therefore be an important element to consider in the management of ME/CFS patients, especially in terms of PEM severity. Subtyping ME/CFS patients according to age would allow identifying a group of patients with higher risk for severe PEM, and therefore needing better adherence to pacing approaches in order to avoid PEM occurrence and prevent exacerbation of the disease.

The second major finding in our study is that recurrent viral infections during the course of the disease were positively associated with PEM severity (OR 2.1 [95% CI 1.2–3.7] (p = 0.009)).

**Fig. 1:**
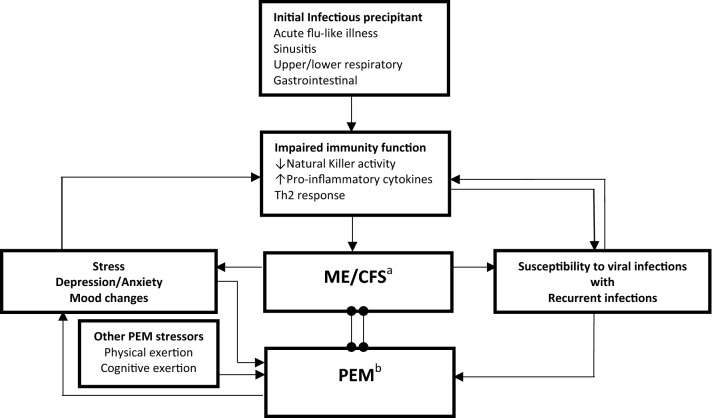
The two-way link between recurrent infections and PEM in ME/CFS patients. An infectious precipitant results in impaired immune function leading to susceptibility to recurrent viral infections and some of ME/CFS manifestations such as fatigue and flu-like symptoms. Stress and mood changes can also impact the immune system. PEM will occur after a stressor (physical, cognitive, emotional and/or infectious) leading to a worsening of ME/CFS baseline symptoms, including immune/inflammatory-related symptoms and psychological disturbances. This will perpetuate the immune dysfunction with aggravation of ME/CFS symptoms, and more frequent viral infections. ^a^ME/CFS: myalgic encephalitis/chronic fatigue syndrome. ^b^PEM: post-exertional malaise

Although the exact ME/CFS pathogenesis is still unknown, there is a growing body of evidence that immune dysfunction elicited by the initial infectious precipitant is one of the main mechanisms involved in ME/CFS [31, 32]. A number of studies reported reduced NK activity making these cells unable to clear viral and other microbial infections, which could explain the susceptibility of these patients to recurrent viral infections [33, 34]. High levels of pro-inflammatory cytokines and a shift towards Th2 response were also reported and seemed to be responsible for some of ME/CFS manifestations such as fatigue and flu-like symptoms [35]. Other factors in ME/CFS patients, such as sleep disturbances, mood changes, and psychological stress can also impact the immune system, thus inducing and/or maintaining immune function abnormalities that contribute to a susceptibility to recurrent, severe, or prolonged viral infections [36]. Faulkner et al. reported frequent recurrence of upper respiratory tract infections in CFS patients compared to healthy controls, and always preceded by high psychological stress and negative mood [37]. Infections were reported to be one of the PEM stressors [10, 12], therefore recurrent infections will result in more frequent and severe PEM and worsening of ME/CFS baseline symptoms. This in turn will perpetuate the immune dysfunction leading to increasing susceptibility to viral infections and symptom severity. It is very likely that the more the patient with ME/CFS has repeated viral infections, the higher the frequency and intensity of his PEM. The 2-way link between recurrent viral infections and PEM severity is illustrated in Fig. 1. Nevertheless, immune function abnormalities are not encountered in all ME/CFS patients and only a subset of patients experience immune/inflammatory-related symptoms. This provides supplementary evidence for the non-homogeneity of the ME/CFS population [34]. The identification of this subset of patients would aid the individualization of patient’s care and allow researchers to find a targeted treatment. Measures against infections, especially viral, and good personal hygiene are highly required among ME/CFS patients with recurrent viral infections, who need to give much more attention to avoiding infections.

The association of recurrent viral infections with severe PEM led us to examine ME/CFS precipitants in our study population, especially infectious precipitants. Amongst the different ME/CFS precipitants, only GI precipitants were associated with more severe PEM (OR 5.7 [1.7–19.3] (p = 0.006)), as shown by an adjusted analysis. A recent study including a large cohort of patients observed an association between all types of GI infections and CFS [38]. Giardia lamblia gastritis [39], gastroenteritis [2], chronic intestinal candidiasis [40], and enteroviruses [41], especially coxsackie viruses [42], were reported to have a causative role in triggering CFS.

Mucosal barrier dysfunction and an increase in gut permeability were reported in ME/CFS resulting in bacterial translocation and consequently a rise in serum endotoxin concentrations, which leads to triggering the immune response [43]. Intestinal microbiota is also found to be altered in ME/CFS patients and thus could contribute to ME/CFS symptoms via increased LPS translocation from gram-negative enterobacteria [44].

GI manifestations are common in ME/CFS and many patients report a previous diagnosis of IBS [45]. Interestingly, both ME/CFS and IBS are sharing many similarities. They may follow bacterial and parasite-induced gastroenteritis, as well as viral disease [39], and gut dysbiosis was reported in both conditions [12]. ME/CFS patients with comorbid IBS may constitute a distinct ME/CFS subgroup, characterized by more severe fatigue and GI symptoms [44]. Dysbiosis could thus trigger autoimmunity, which in turn might be responsible for mitochondrial dysfunction identified as having a role not only in PEM pathophysiology [12, 13], but also in terms of PEM severity as we have recently demonstrated [14].

Consequently, ME/CFS-related GI anomalies could explain increasing PEM severity observed in patients in whom the disease onset was preceded by the occurrence of GI infection.

## Limitations and strengths

The statistically significant association observed between GI infectious precipitants and PEM severity must be interpreted with caution due to the small number of patients in whom the ME/CFS was preceded by GI infectious event. The multivariate analysis of factors associated with PEM severity included variables that were probably statistically underpowered (neurosensory, perceptual and motor disturbances, and sleep disturbances) and produced wide confidence interval. PEM assessment could be biased by the subjectivity of the used instrument and it would be better to support this assessment with an objective evaluation. At present, the only available objective instrument is the 2-day cardiopulmonary exercise test (CPET) that objectively demonstrates the loss of function and lack of recovery that occurs following exertion. However, the CPET carries substantial risk for patients as it may worsen their condition by triggering PEM. In addition, its systematic use in the research field is limited because of cost, expertise, and the level of severity of some participants [46]. Another source of weakness was the retrospective character of data collection and the lack of information concerning the mode of onset of the disease.

On the other hand, we would like to highlight the sizeable number of the study population and the fact that all patients were examined and diagnosed by the same physician, and underwent a same standardized procedure in terms of PEM and fatigue assessments. Clinical manifestations were studied on the basis of symptom patterns according to the ME ICC criteria [3]. It is also of interest to note that the statistical method was planned a priori and considered all variables without any selection.

## Conclusion

Given the non-homogeneity of the ME/CFS population, and knowing that PEM is associated with disability and poorer outcome, we attempted to identify patients with higher risk for severe PEM on epidemiological and clinical features. To the best of our knowledge, our study is the first to identify factors that may influence PEM severity in ME/CFS patients.

We observed more severe PEM in older patients at disease onset, and among those who were suffering from recurrent infections during their disease course. More severe PEM was also observed in patients in whom ME/CFS onset was preceded by GI infectious precipitants.

Accordingly, this will allow adapting and individualizing the disease management, especially in the absence of curative treatment. Hence older patients should be advised to adhere more strictly to pacing strategies, and specific measures against infections together with pacing should be recommended for those who display recurrent and/or persistent infections. The aim is to prevent PEM occurrence, or at least reducing its severity, to help improving disease course and patients’ quality of life.

## Data Availability

The datasets used and/or analyzed during the current study are available from the corresponding author on reasonable request.
